# Serum TIMP-1 shows a potential association with metastatic disease in patients with pancreatic cancer: a pilot analysis without a healthy control cohort

**DOI:** 10.1007/s10238-026-02241-0

**Published:** 2026-06-27

**Authors:** Ivana Vecurkovska, Veronika Roskovicova, Tomas Gajdzik, Marek Soltes, Jana Maslankova, Ivana Kocanova, Jana Katuchova

**Affiliations:** 1 Department of Medical and Clinical Biochemistry, Medical Faculty, Pavol Jozef Šafárik University in Košice, Košice, Slovak Republic; 2 Department of Technical Disciplines in Health Care, University of Prešov, Prešov, Slovakia; 3 1st Department of Surgery, Medical Faculty, University of Pavol Jozef Šafárik in Košice and Louis Pasteur University Hospital in Košice, Trieda SNP 1, 040 0, Košice, Slovak Republic

**Keywords:** TIMP-1, Pancreatic cancer, Metastases, Biomarker

## Abstract

Pancreatic ductal adenocarcinoma (PDAC) is most often diagnosed at an advanced stage, when distant metastases are already present and curative treatment options are limited. In this clinical context, biomarkers capable of reflecting tumor aggressiveness and metastatic potential are of particular relevance. Tissue inhibitor of metalloproteinases-1 (TIMP-1) has been implicated in tumor invasion and metastatic dissemination; however, its clinical significance in pancreatic cancer remains incompletely defined. We analyzed TIMP-1 concentrations in serum and pancreatic tissue of 154 patients (108 malignant tumors, 46 benign lesions) using ELISA. Associations with clinical stage and the presence of distant metastases were assessed using non-parametric tests and ROC curve analysis. TIMP-1 levels were significantly higher in malignant compared with benign tumors, both in tissue (24.28 vs. 21.62 ng/mL; *p* = 0.0318) and serum (28.03 vs. 25.52 ng/mL; *p* = 0.0106). Serum TIMP-1 showed superior diagnostic accuracy (AUC = 0.796) compared to tissue TIMP-1 (AUC = 0.745). Patients with distant metastases exhibited significantly higher serum TIMP-1 levels than non-metastatic cases (28.33 vs. 26.53 ng/mL; *p* = 0.0046; AUC = 0.785). Serum TIMP-1 is associated with malignant and metastatic pancreatic disease and reflects a more aggressive tumor phenotype in patients with established pancreatic cancer. While the observed differences are modest and limit its applicability as a stand-alone diagnostic or screening biomarker, TIMP-1 may provide clinically relevant information regarding metastatic status and could contribute to prognostic stratification or multimarker approaches in pancreatic cancer.

## Introduction

Pancreatic ductal adenocarcinoma (PDAC) is among the most aggressive and prognostically unfavourable malignancies, with a five-year survival rate that remains dismal despite advances in diagnostic and therapeutic strategies [1, 2]. A fundamental challenge in the management of pancreatic cancer is its late clinical presentation; most patients are diagnosed at an advanced stage, frequently with locally invasive or metastatic disease, which severely limits curative treatment options [3]. Consequently, improvements in patient outcomes depend not only on earlier detection—which remains difficult to achieve—but also on more accurate stratification of patients according to tumor aggressiveness, metastatic potential, and expected clinical course at the time of diagnosis.

Current diagnostic approaches, including computed tomography (CT), magnetic resonance imaging (MRI), and endoscopic ultrasonography with fine-needle aspiration (EUS-FNA), are essential for tumor detection and staging but provide limited information regarding the biological behaviour of the tumor [3, 4]. In this context, circulating biomarkers may offer complementary insights by reflecting systemic and tumor-derived processes associated with invasion, dissemination, and metastatic spread. Rather than serving as screening tools, such biomarkers may be particularly valuable for identifying biologically aggressive disease, guiding therapeutic decision-making, and supporting prognostic stratification in patients with established pancreatic cancer.

Matrix metalloproteinases (MMPs) constitute a family of zinc-dependent endopeptidases that play a central role in extracellular matrix remodelling, tumor invasion, angiogenesis, and metastasis [5]. Their proteolytic activity is tightly regulated by tissue inhibitors of metalloproteinases (TIMPs), among which tissue inhibitor of metalloproteinases-1 (TIMP-1) is one of the most extensively studied [5, 6]. Dysregulation of the MMP–TIMP balance has been implicated in the progression of multiple malignancies, including pancreatic cancer, where enhanced matrix remodelling facilitates local invasion and distant tumor spread [7, 8].

Although TIMP-1 was initially characterized as an endogenous inhibitor of MMPs, accumulating evidence suggests that its role in cancer biology extends beyond protease inhibition. TIMP-1 has been shown to influence cell proliferation, survival, immune modulation, and the formation of pre-metastatic niches through MMP-independent mechanisms [8]. In pancreatic cancer, elevated TIMP-1 expression has been associated with invasive growth patterns, venous invasion, and liver metastasis, highlighting its potential relevance as a marker of metastatic tumor biology rather than an indicator of early tumor presence [9–11].

The clinical significance of circulating TIMP-1 levels in pancreatic cancer, however, remains incompletely defined. While previous studies have reported increased TIMP-1 concentrations in patients with PDAC, its diagnostic performance as a stand-alone biomarker has been limited, and its greatest potential appears to lie in reflecting tumor aggressiveness or contributing to multimarker panels rather than in early detection [12, 13]. Moreover, data directly linking serum TIMP-1 levels to metastatic status in clinically characterized patient cohorts are still scarce.

Therefore, the aim of the present study was to investigate serum and tissue concentrations of TIMP-1 in patients with pancreatic tumors, with a particular focus on their association with malignant disease and the presence of distant metastases. Specifically, we sought to (i) compare TIMP-1 levels between benign and malignant pancreatic lesions, (ii) evaluate their relationship with disease stage, and (iii) assess the ability of serum TIMP-1 to discriminate between patients with and without metastatic disease. By framing TIMP-1 within the context of metastatic biology rather than early diagnosis, this study aims to contribute to a more precise understanding of its potential clinical relevance in pancreatic cancer.

## Materials and methods

The basic demographic characteristics of the patients are summarized in Table 1. A total of 108 subjects in the malignant tumor group (MTG; Stage I: 11; II: 23; III: 31; IV: 43) and 46 subjects in the group with benign pancreatic findings (BTG) were included in the analysis. Patients were divided into groups based on histological examination of tissue samples and the standard TNM classification. Patients with confirmed chronic pancreatitis, pancreatic pseudocyst, and serous microcystic adenoma were included in the BTG. The presence or absence of metastases was determined by imaging (CT, MRI) or histology.

In the gender analysis, there was no statistically significant difference in distribution between the groups (MTG: 48.1% female, 51.9% male; BTG: 34.8% female, 65.2% male; Chi-square test, *p* = 0.072). However, we found a statistically significant difference in age between groups (MTG: median 68 years; BTG: median 63 years; Mann-Whitney U test, *p* = 0.001). Specifically, the MTG group had a significantly higher proportion of subjects aged 50 or older (94.5%) than the BTG group (73.9%), indicating that the MTG group is, on average, older than the BTG group. Patients included in this study were not treated with any form of cancer therapy.

**Table 1:** The basic demographic characteristics of patients

	MTG 108 (70.1%)	BTG 46 (29.9%)	Sig.
**Gender**			
Female	52 (48.1%)	16 (34.8%)	0.072
Male	56 (51.9%)	30 (65.2%)	
**Age**			
Median	68	63	**0.001**
<50 years	6 (5.5%)	12 (26.1%)	
>50 years	102 (94.5%)	34 (73.9%)	

The collection of biological material, specifically blood serum and pancreatic tissue, was conducted at the 1st Department of Surgery, Faculty of Medicine, Pavol Jozef Šafárik University, and Louis Pasteur University Hospital in Košice in the period September 2022-December 2024. Patients scheduled for pancreatic surgery were recruited for this study. Before participation, all patients received comprehensive information about the study and provided written informed consent, in accordance with the provisions of Sect.  27 of Act no. 576/2004 on healthcare. The research was approved by the Ethics Committee of UNLP in Košice under approval number 2024/EK/03041.

Blood samples were obtained preoperatively in the morning from fasting patients. Venous blood was collected into BD Vacutainer tubes containing a separation gel. For a subset of patients, pancreatic tissue samples were collected intraoperatively. All collected biological samples were immediately transferred to the Department of Medical and Clinical Biochemistry for subsequent processing, long-term storage, and biochemical analysis.

Laboratory processing involved centrifuging whole blood at 3,500 x g for 10 min at room temperature to obtain serum. Tissue samples were first rinsed with phosphate-buffered saline (PBS) to remove contaminants, then homogenized in an appropriate extraction buffer on ice, and finally centrifuged at 18,000 x g for 20 min at 4 °C to obtain the desired supernatant.

TIMP-1 concentrations in blood serum and pancreatic tissue were measured by ELISA (Human TIMP-1 ELISA Kit, EH456RB, Invitrogen) according to the manufacturer’s instructions. The samples were analyzed in duplicates. Absorbance was measured using an Elx808 Microplate Reader (Biotech, Bratislava, Slovakia) at 450 nm, and the concentration was calculated from the standard curve. TIMP-1 concentrations were expressed in ng/mL.

Descriptive statistics and Spearman correlation tests were performed using IBM SPSS 23 Statistics. All other statistical analyses were conducted in GraphPad Prism. The normality of data distribution was assessed using the Shapiro–Wilk test. Since all variables showed non-normal distribution, comparisons between two groups were performed using the Mann–Whitney U test, while Dunn’s multiple comparison test was applied for analyses involving more than two groups. For statistically significant results, receiver operating characteristic (ROC) curves were constructed to evaluate the discriminative ability of selected parameters. A p-value < 0.05 was considered statistically significant.

## Results

Spearman correlation analysis revealed several statistically significant relationships between patient variables (Table 2).

**Table 2:** Spearman correlation analysis between patient variables

		Gender	Age	Group	Stage
*Gender*	Correlation Coefficient	1	0.186^*^	-0.143	-0.127
	Sig. (2-tailed)	NA	**0.021**	0.076	0.117
	N	154	**154**	154	154
*Age*	Correlation Coefficient	**0.186** ^*^	1	**-0.338** ^**^	**-0.254** ^**^
	Sig. (2-tailed)	**0.021**	NA	**0**	**0.002**
	N	**154**	154	**154**	**154**
*Group*	Correlation Coefficient	-0.143	**-0.338** ^**^	1	**0.827** ^**^
	Sig. (2-tailed)	0.076	**0**	NA	**0**
	N	154	**154**	154	**154**
*Stage*	Correlation Coefficient	-0.127	**-0.254** ^**^	**0.827** ^**^	1
	Sig. (2-tailed)	0.117	**0.002**	**0**	NA
	N	154	**154**	**154**	154

*. Correlation is significant at the 0.05 level (2-tailed)

**. Correlation is significant at the 0.01 level (2-tailed)

NA: p-values are not applicable for correlations of variables with themselves (diagonal)

Gender: 1-Female, 2-Male; Age: 1->50, 2-<50; Group: 1-BTG, 2-MTG; Stage: 0-BTG. 1-Stage I, 2-Stage II, 3-Stage III, 4-Stage IV

A significant positive correlation was observed between patient age and gender (*r* = 0.186; *p* = 0.021), with older age more common among men. Age was also negatively correlated with MTG group membership (*r*=-0.338; *p* < 0.001), indicating a higher incidence of malignant tumors in older patients. In addition, higher stages were statistically significantly more common in older patients (*r*=-0.254; *p* = 0.002).

Our study focused on the quantification of TIMP-1, which plays a key role in ECM remodeling and cancer progression. Initial analysis was primarily focused on characterizing the expression of these molecules directly in excised pancreatic tissue samples, providing us with a detailed view of local pathological processes. However, to contribute to the development of less invasive methods that minimize patient discomfort and risks, we subsequently extended our analysis to blood serum samples. We hypothesized that changes in the expression of these biomarkers in tumor tissue might also be reflected in the systemic circulation, thus offering potential for use in disease monitoring. A notable finding of our study is that serum analysis demonstrated stronger statistical significance, reflected by lower p-values and more pronounced discrimination between the studied groups, compared to tissue analysis. This comparison is innovative because it evaluates the biomarker in both tissue and serum within the same cohort, highlighting its potential use in minimally invasive diagnostics.

**Fig. 1:**
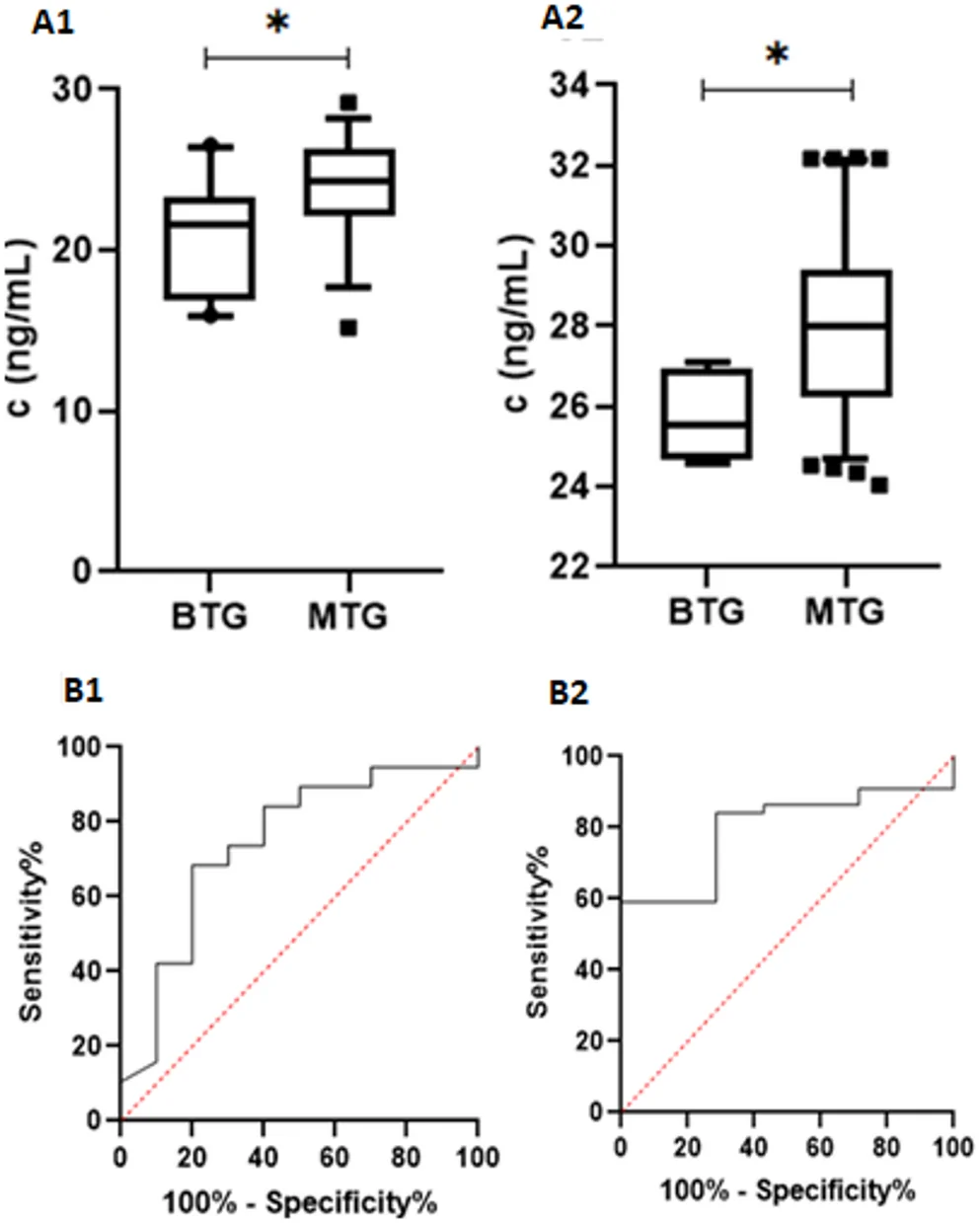
Boxplots for TIMP-1 in tissue (A1) and in serum (A2), between BTG and MTG patients. ROC curves for TIMP-1 in tissue (B1) and in serum (B2) for patients in BTG and MTG

Table 3 summarizes the distribution of TIMP-1 values measured in both tissue and serum samples from patients divided into BTG and MTG. Median concentrations (ng/mL) for each molecule analysed and sample type are provided, along with the 25th and 75th percentiles, which define the interquartile range and serve as a measure of data scatter. The data are graphically processed in Fig. 1.

**Table 3:** Tissue and serum concentrations (median, 25th and 75th percentile) of TIMP-1 in BTG and MTG

	Median (ng/mL)	25% Percentile	75% Percentile	Sig.
Tissue TIMP-1				
BTG	21.62	16.84	23.37	**0.0318**
MTG	24.28	22.14	26.33	
***Serum TIMP-1***				
BTG	25.52	24.67	26.97	**0.0106**
MTG	28.03	26.23	29.43	

The data presented show that TIMP-1 has significantly higher median concentrations in patients with MTG than in those with BTG, both in tissue (21.62 vs. 24.28 ng/mL; *p* = 0.0318) and in serum (25.52 vs. 28.03 ng/mL; *p* = 0.0106).

To further characterize the potential of this biomarker and to determine optimal cut-off values and quantify their ability to discriminate between BTG and MTG, we subsequently generated ROC curves. Analysis of the ROC curves allowed us to comprehensively assess the sensitivity and specificity of each biomarker at different thresholds and thus identify their accuracy. Specifically, the area under the curve (AUC) provides an overall measure of a biomarker’s discriminative power, while analysis of the ROC curve coordinates allows us to determine optimal cut-off points for maximum diagnostic efficiency (Fig. 1).

Table 4 presents the results of the ROC analysis for TIMP-1 in tissue and serum samples. This biomarker analyzed showed statistically significant discriminatory ability (*p* < 0.05). The data are graphically processed in Fig. 1.

**Table 4:** Results of ROC curve analysis for the diagnostic accuracy of TIMP-1 in tissue and serum samples

	AUC	Std. Error	95% CI	Sig.
Tissue TIMP-1	0.7447	0.1009	0.5470–0.9425	0.0329
*Serum TIMP-1*	0.7955	0.0695	0.6593–0.9316	**0.0127**

The ROC analysis revealed that serum TIMP-1 had greater discriminative ability (AUC = 0.7955) than tissue TIMP-1 (AUC = 0.7447). This indicates that serum TIMP-1 provides better diagnostic accuracy, with a statistically more significant association (lower p-value). Optimal cut-off value was 27.15 ng/mL (sensitivity 59.09%, specificity 100.0%; J = 59.09%) for serum, and 23.28 ng/mL (sensitivity 68.42%, specificity 80.0%; J = 48.42%) for tissue.

To investigate the relationship between the concentrations of the analyzed biomarker and disease progression, we subsequently analyzed the serum levels of TIMP-1 concerning the different clinical stages of pancreatic malignancy (Stage I, II, III, IV), comparing them with BTG. We found that the median TIMP-1 value tends to increase with disease progression, with the greatest increase and the most significant differences observed between the BTG group and stage IV patients.

Serum TIMP-1 concentrations were significantly higher in patients with stage IV malignancy compared with the benign group (28.33 ng/mL vs. 25.52 ng/mL, *p* = 0.0122), and ROC analysis demonstrated excellent discriminatory performance of TIMP-1 for distinguishing these groups (AUC = 0.9481, *p* = 0.0018). Since the most statistically significant differences were observed compared with patients in stage IV, which is associated with distant metastases, our attention was further focused on the key clinical question. Rather than strict staging, which requires a comprehensive diagnostic workup, we decided to prioritize exploring the diagnostic ability of this serum biomarker in differentiating patients with metastases present from those without metastases. We aimed to identify a simple, non-invasive method to reliably differentiate metastatic disease, as its detection is crucial for patient stratification and therapeutic decision-making.

**Fig. 2:**
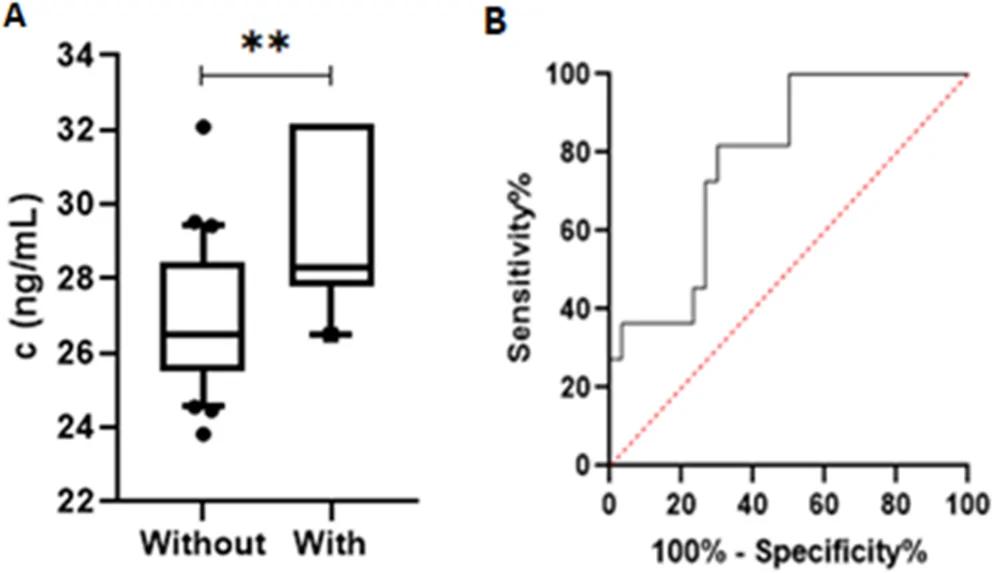
Boxplots for serum TIMP-1 (**A**) for patients without and with distant metastases. ROC curves for TIMP-1 (**B**) for patients without or with distant metastases

Table 5 presents the median concentrations and interquartile ranges of serum TIMP-1 in a group of patients stratified according to the presence (“With”) or absence (“Without”) of distant metastases. The data are graphically processed in Fig. 2.

**Table 5:** Serum concentrations (median, 25th and 75th percentile) of TIMP-1 in patients without and with distant metastases

	Median (ng/mL)	25% Percentile	75% Percentile	Sig.
Without With	26.53 28.33	25.50 27.80	28.43 32.18	**0.0046**

The results clearly demonstrate that TIMP-1 concentrations are statistically significantly higher in patients with metastases than in those without (*p* < 0.01). Serum TIMP-1 showed an increase from 26.53 ng/mL to 28.33 ng/mL (*p* = 0.0046).

Table 6 summarizes the results of ROC analysis for serum concentrations of TIMP-1, focusing on their ability to predict the presence of metastasis. The data are graphically processed in Fig. 2.

**Table 6:** Results of ROC curve analysis for the diagnostic accuracy of TIMP-1 in patients without and with distant metastases

	Area	Std. Error	95% CI	Sig.
*Serum TIMP-1*	0.7848	0.0739	0.6399–0.9298	**0.0057**

ROC analysis demonstrated a moderate discriminatory ability of serum TIMP-1 for metastatic disease (AUC = 0.7848, *p* = 0.0057). The optimal cut-off value according to Youden’s index (52.05%) was 27.77 ng/mL (sensitivity 81.8%, specificity 70.0%).

## Discussion

Pancreatic ductal adenocarcinoma remains one of the deadliest malignancies, largely due to its silent progression and late clinical presentation. As most patients are diagnosed at an advanced stage, frequently with distant metastases already present, the clinical utility of biomarkers in pancreatic cancer differs fundamentally from that in malignancies amenable to population screening. In this context, biomarkers that reflect tumor aggressiveness, metastatic competence, or biological behavior at the time of diagnosis may be of greater clinical relevance than markers aimed at early detection.

In contrast to previous studies that primarily examined TIMP-1 as a general diagnostic biomarker, the novelty of our work lies in demonstrating its specific association with metastatic disease, supported by a dual analysis of serum and tumor tissue within the same patient cohort and by positioning TIMP-1 within a clinically relevant framework of stratifying metastatic versus non-metastatic pancreatic cancer.

In this study, we observed significantly higher TIMP-1 concentrations in patients with malignant pancreatic tumors compared with those with benign pancreatic lesions, both in tissue and in serum. Importantly, serum TIMP-1 demonstrated superior discriminative ability compared with tissue levels, underscoring the potential of circulating biomarkers to reflect systemic tumor-relatedco processes. Although the observed differences in TIMP-1 concentrations between benign and malignant groups were modest, they followed a consistent directional trend and were statistically significant within our cohort. However, given the small effect size, TIMP-1 alone may have limited diagnostic utility and should be interpreted with caution, ideally in combination with other biomarkers and validated in larger independent cohorts.

The most clinically relevant finding of this study is the significant association between elevated serum TIMP-1 levels and the presence of distant metastases. Patients with metastatic disease exhibited higher serum TIMP-1 concentrations than non-metastatic patients, with good discriminatory performance, as reflected by ROC analysis. From a clinical perspective, such information may be valuable for patient stratification, prognostic assessment, and therapeutic decision-making in individuals with established pancreatic cancer.

These findings are consistent with accumulating experimental and clinical evidence indicating that TIMP-1 plays an active role in pancreatic cancer progression beyond its classical function as an inhibitor of matrix metalloproteinases [9, 14–16]. TIMP-1 may promote liver metastasis via MMP-independent mechanisms, including CD63-mediated activation of hepatic stellate cells and the establishment of a pre-metastatic niche. Moreover, tumor-derived TIMP-1 may contribute to venous invasion, immune modulation, and the activation of oncogenic signaling pathways such as PI3K/AKT and ERK1/2.

Together, these data support the interpretation of circulating TIMP-1 as a marker of invasive and metastatic tumor behavior rather than a surrogate of tumor burden alone [10, 11].

The relatively subtle differences in TIMP-1 concentrations observed between the individual groups in this study highlight an important limitation of TIMP-1 as a stand-alone biomarker. Such effect sizes are unlikely to be sufficient to support its use as a screening tool or as a single diagnostic marker in clinical practice. This limitation is further compounded by the absence of a healthy control group, which precludes the establishment of a physiological baseline for circulating TIMP-1 levels and a direct assessment of whether the observed concentrations are elevated compared with the general population.

Therefore, our findings should primarily be interpreted as relative differences within a cohort of surgically treated patients rather than as evidence of an absolute increase in TIMP-1 above normal population values. In the future, we plan to expand the study cohort to include healthy controls, which will allow a more precise definition of reference TIMP-1 levels and a better assessment of its potential diagnostic value.

Nevertheless, the current study design intentionally reflects a real-world clinical context, in which clinicians are often not faced with the need to distinguish patients from completely healthy individuals, but rather with the need to correctly characterize lesions in patients already undergoing diagnostic evaluation or surgical treatment. In this setting, the greatest diagnostic challenge lies particularly in distinguishing benign from malignant lesions, as well as in identifying more aggressive or metastatic forms of disease. From this perspective, our findings may remain clinically relevant despite the absence of a healthy control group, as they point to a potential role of TIMP-1 in differentiating patients within routine clinical practice.

Notably, previous studies have demonstrated that while TIMP-1 alone performs less favorably than established markers such as CA19-9, its inclusion in multimarker panels substantially improves diagnostic and prognostic accuracy. In this context, TIMP-1 may contribute complementary biological information related to tumor invasiveness and metastatic potential, thereby enhancing the performance of composite biomarker signatures [12, 13, 17]. Multimodal profiling of PDAC has shown that TIMP-1 is part of a dominant secretory phenotype associated with more aggressive tumors and immunomodulatory changes, supporting its role as a functional biomarker [18]. A 2023 meta-analysis of protein biomarkers further demonstrated that novel protein markers, including TIMP-1, provide added diagnostic value when combined with CA19-9, with multimarker models outperforming CA19-9 alone [19]. This trend is reinforced by validation studies aimed at early PDAC detection, where protein panels incorporating TIMP-1 showed improved accuracy in identifying early-stage disease [20]. Moreover, recent work integrating protein biomarkers with alternatively methylated circulating DNA demonstrated that multimarker assays can surpass the performance of CA19-9 alone, with TIMP-1 contributing to improved robustness across clinical subgroups [21]. Collectively, these findings support the interpretation that although TIMP-1 has limited value as a standalone marker, it provides significant added benefit within multimarker diagnostic panels, which may offer greater accuracy for PDAC detection than CA19-9 alone. Our findings support this concept by demonstrating a reproducible association between serum TIMP-1 and metastatic disease, reinforcing its potential role within integrated biomarker approaches rather than as an isolated clinical test.

From a future perspective, the role of TIMP‑1 in pancreatic cancer should be explored primarily within the framework of multimarker strategies rather than as a standalone diagnostic test. Given its association with more advanced and biologically aggressive disease, TIMP‑1 appears particularly promising as a component of composite panels that integrate established markers such as CA19‑9 with additional protein biomarkers, imaging findings, and possibly circulating tumor DNA. Future studies should therefore aim to validate TIMP‑1‑containing panels in large, prospectively collected cohorts that include both resectable and unresectable cases, as well as patients across all disease stages, to better capture the full clinical spectrum. It will also be important to assess whether TIMP‑1 can improve risk stratification, for example by identifying patients at higher likelihood of occult metastases or early recurrence after surgery. In addition, longitudinal studies evaluating dynamic changes in TIMP‑1 levels during treatment and follow‑up could clarify its potential utility as a marker of treatment response, minimal residual disease or survival using Kaplan-Meier curves. Ultimately, integrating TIMP‑1 into robust, clinically applicable multimarker algorithms, rather than evaluating it in isolation, may offer the most realistic path toward improving personalized management of pancreatic ductal adenocarcinoma. Although TIMP-1 is known to participate in biological processes such as CAF activation and EMT, we did not perform molecular or genetic profiling of tumor tissue. In future research, integrating serum TIMP-1 measurements with specific genetic and molecular markers could substantially improve patient stratification by identifying subgroups in whom TIMP-1 monitoring may carry the greatest clinical relevance, particularly in relation to metastatic potential and tumor aggressiveness.

Several limitations of this study warrant consideration. The study cohort was relatively limited, which represents an important constraint, particularly for subgroup analyses according to disease stage and metastatic status. Therefore, the results of these stratified analyses should be interpreted with caution and considered exploratory rather than definitive. The cut-off values derived from our ROC analysis should likewise be interpreted within the context of our specific study cohort, and their clinical applicability requires further validation in larger, independent populations before routine implementation. In addition to the modest cohort size and absence of healthy controls, demographic differences between the malignant and benign groups, particularly age, may have influenced TIMP-1 concentrations. Moreover, TIMP-1 levels showed the greatest differences in patients with advanced or metastatic disease, suggesting limited sensitivity for distinguishing early disease stages. These limitations underscore the need for validation in larger, multicenter cohorts and for studies incorporating longitudinal sampling and multimarker analyses. At the same time, future studies should also include patients who do not undergo pancreatic surgery, which would help avoid the selection bias introduced by excluding individuals with non-operable or advanced disease and allow for a more comprehensive assessment across the full clinical spectrum.

Another limitation of this study is the age imbalance between the benign and malignant groups, which may represent a potential confounding factor influencing TIMP-1 concentrations. This difference, however, partly reflects the known epidemiology of pancreatic diseases. Benign pancreatic lesions, such as serous cystadenoma or pancreatic pseudocysts associated with chronic pancreatitis, may occur across a broader age range and are also observed in younger patients. In contrast, pancreatic ductal adenocarcinoma is predominantly a disease of older age, with the highest incidence reported in patients over 65 years. This pattern was also observed in our cohort, where the median age of patients with malignant pancreatic tumors was 68 years, while the median age in the benign group was 63 years, consistent with the reported peak occurrence of many benign pancreatic lesions between 50 and 70 years of age. Nevertheless, the potential influence of age on TIMP-1 levels cannot be excluded. Therefore, our findings should be interpreted with caution, and future studies involving larger, age-balanced cohorts and age-adjusted analyses are needed to confirm whether TIMP-1 is independently associated with malignant and metastatic pancreatic disease.

In summary, the present study demonstrates that serum TIMP-1 is associated with malignant and metastatic pancreatic disease and reflects biologically aggressive tumor behavior in patients with established pancreatic cancer. While its utility for early detection or stand-alone diagnosis is limited, TIMP-1 provides insight into metastatic biology and may contribute to prognostic stratification and multimarker strategies aimed at improving the clinical management of pancreatic cancer.

## Conclusions

In this study, serum and tissue TIMP-1 concentrations differed between malignant and benign pancreatic lesions, with serum levels showing a stronger association with disease status. Higher serum TIMP-1 levels were observed in patients with distant metastases, indicating a possible association with metastatic disease. Nevertheless, the magnitude of these differences was modest, and the limited cohort size, particularly in subgroup analyses, restricts the strength and generalizability of these findings. Accordingly, the present results do not support the use of TIMP-1 as a stand-alone diagnostic or screening biomarker. TIMP-1 may, however, represent a complementary biomarker within multimarker strategies aimed at improving risk stratification in patients with established pancreatic cancer. Larger prospective studies, including balanced cohorts, adjusted analyses and survival endpoints, are needed to determine whether TIMP-1 has independent prognostic or clinical utility.

## Data Availability

No datasets were generated or analysed during the current study.
